# Poly[[(μ_2_-acetato-κ^3^ *O*,*O*′:*O*′)aqua­bis­(μ_3_-isonicotinato-κ^3^ *O*:*O*′:*N*)samarium(III)silver(I)] perchlorate]

**DOI:** 10.1107/S1600536811026134

**Published:** 2011-07-09

**Authors:** Li-Cai Zhu, Si-Ming Zhu

**Affiliations:** a School of Chemistry and Environment, South China Normal University, Guangzhou 510631, People’s Republic of China; b School of Light Industry and Food Science, South China University of Technology, Guangzhou 510641, People’s Republic of China

## Abstract

The title compound, {[AgSm(C_6_H_4_NO_2_)_2_(CH_3_CO_2_)(H_2_O)]ClO_4_}_*n*_, is a three-dimensional heterobimetallic complex constructed from a repeating dimeric unit. Only half of the dimeric moiety is found in the asymmetric unit; the unit cell is completed by crystallographic inversion symmetry. The Sm^III^ ion is eight-coordinated by four O atoms of four different isonicotinate ligands, three O atoms of two different acetate ligands, and one O atom of a water mol­ecule. The two-coordinate Ag^I^ ion is bonded to two N atoms of two different isonicotinate anions, thereby connecting the disamarium units. In addition, the isonicotinate ligands also act as bridging ligands, generating a three-dimensional network. The coordinated water mol­ecules link the carboxyl­ate group and acetate ligands by O—H⋯O hydrogen bonding. Another O—H⋯O hydrogen bond is observed in the crystal structure. The perchlorate ion is disordered over two sites with site-occupancy factors of 0.560 (11) and 0.440 (11), whereas the methyl group of the acetate ligand is disordered over two sites with site-occupancy factors of 0.53 (5) and 0.47 (5).

## Related literature

For background to lanthanide–transition metal heterometallic complexes, see: Cheng *et al.* (2006 ▶); Kuang *et al.* (2007 ▶); Peng *et al.* (2008 ▶); Zhu *et al.* (2009 ▶). 
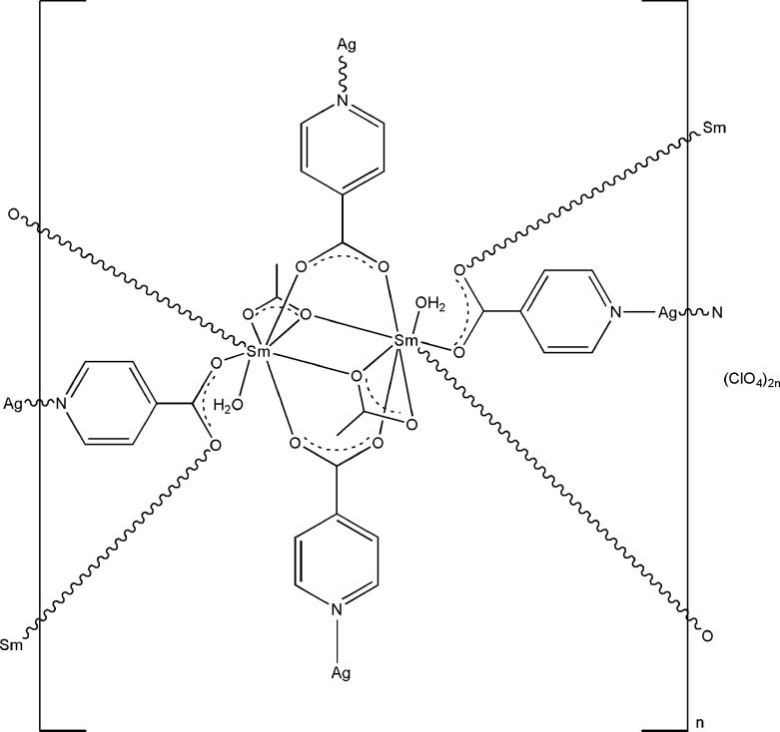


## Experimental

### 

#### Crystal data

[AgSm(C_6_H_4_NO_2_)_2_(C_2_H_3_O_2_)(H_2_O)]ClO_4_
*M* *_r_* = 678.94
Monoclinic, 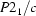

*a* = 16.1703 (15) Å
*b* = 15.1042 (14) Å
*c* = 7.9858 (7) Å
β = 92.845 (1)°
*V* = 1948.0 (3) Å^3^
*Z* = 4
Mo *K*α radiation
μ = 4.19 mm^−1^
*T* = 296 K
0.22 × 0.20 × 0.19 mm

#### Data collection

Bruker APEXII area-detector diffractometer
Absorption correction: multi-scan (*SADABS*; Sheldrick, 1996 ▶) *T*_min_ = 0.414, *T*_max_ = 0.451
9927 measured reflections
3512 independent reflections
2957 reflections with *I* > 2σ(*I*)
*R*_int_ = 0.030

#### Refinement

*R*[*F* ^2^ > 2σ(*F* ^2^)] = 0.026
*wR*(*F* ^2^) = 0.064
*S* = 1.07
3512 reflections
320 parameters
158 restraints
H atoms treated by a mixture of independent and constrained refinement
Δρ_max_ = 0.85 e Å^−3^
Δρ_min_ = −0.59 e Å^−3^

### 

Data collection: *APEX2* (Bruker, 2004 ▶); cell refinement: *SAINT* (Bruker, 2004 ▶); data reduction: *SAINT*; program(s) used to solve structure: *SHELXS97* (Sheldrick, 2008 ▶); program(s) used to refine structure: *SHELXL97* (Sheldrick, 2008 ▶); molecular graphics: *SHELXTL* (Sheldrick, 2008 ▶); software used to prepare material for publication: *SHELXTL*.

## Supplementary Material

Crystal structure: contains datablock(s) I, global. DOI: 10.1107/S1600536811026134/im2297sup1.cif

Structure factors: contains datablock(s) I. DOI: 10.1107/S1600536811026134/im2297Isup2.hkl

Additional supplementary materials:  crystallographic information; 3D view; checkCIF report

## Figures and Tables

**Table 1:** Hydrogen-bond geometry (Å, °)

*D*—H⋯*A*	*D*—H	H⋯*A*	*D*⋯*A*	*D*—H⋯*A*
O1*W*—H2*W*⋯O6^i^	0.81 (4)	1.98 (4)	2.785 (4)	171 (6)
O1*W*—H1*W*⋯O2^ii^	0.81 (4)	2.22 (3)	2.921 (5)	146 (5)

Symmetry codes: (i) 
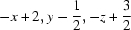
; (ii) 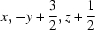.

**Fig. 1.**
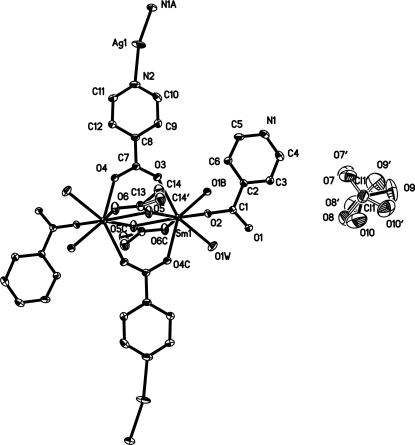
Molecular structure showing displacement ellipsoids drawn at the 30% probability level. Hydrogen atoms are omitted for clarity. Symmetry codes: (A) 1 - x, 1/2 + y, 2.5 - z; (B) x, 1.5 - y, 1/2 + z; (C) 2 - x, 2 - y, 2 - z.

**Fig. 2.**
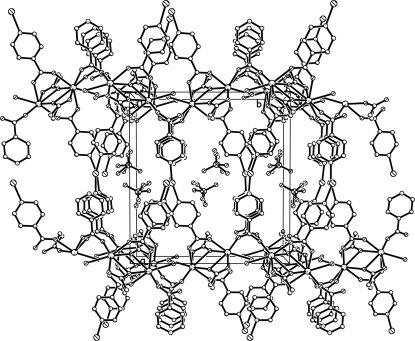
A view of the three-dimensional structure of the title compound. Hydrogen atoms are omitted for clarity.

## supplementary crystallographic information

### Comment

In the past few years, lanthanide-transition metal heterometallic complexes with bridging multifunctional organic ligands are of increasing interest not only because of their impressive topological structures, but also due to their versatile applications in ion exchange, magnetism, bimetallic catalysis and as luminescent probes (Cheng *et al.*, 2006; Kuang *et al.*, 2007; Peng *et al.*, 2008; Zhu *et al.*, 2009). As an extension of this research, the structure of the title compound, a new heterobimetallic coordination polymer, (I), has been determined which is presented in this artcle.

The polymer of the title compound displays a three-dimensional heterometallic coordination framework constructed from the repeating dimeric unit. Only half of the dimeric unit is found in the asymmetric unit whose center is a crystallographic center of inversion (Fig. 1). The asymmetric unit of the title compound, contains one of the Sm^III^ and Ag^I^ ions each, two halves of the acetate ligands, two isonicotinate ligands and one coordinated water molecule. The Sm^III^ ion is eight-coordinated by four O atoms of four different isonicotinate ligands, three O atoms of two different acetate ligands and one O atom of a water molecule. The Sm center can therefore be described as adopting a bicapped trigonal prismatic coordination geometry. The two-coordinate Ag^I^ ion is bonded to two N atoms of two different isonicotinate anions. The Ag^I^ ion shows an almost linear coordination with N1—Ag1—N2 being 166.2 (2) °. These metal coordination units are additionally connected by bridging isonicotinate and acetate ligands, generating a three-dimensional network (Fig. 2).The coordinated water molecules link the carboxylate group and acetate ligand by O—H···O hydrogen bonding (Table 1). Another O–H···O hydrogen bond is observed in the crystal structure.

### Experimental

A mixture of AgNO_3 _(0.057 g, 0.33 mmol), Sm_2_O_3 _(0.116 g, 0.33 mmol), isonicotinic acid (0.164 g, 1.33 mmol), CH_3_COONa (0.057 g, 0.7 mmol), H_2_O (7 ml), and HClO_4 _(0.257 mmol, pH 2) was sealed in a 20 ml Teflon-lined reaction vessel at 443 K for 6 days then slowly cooled to room temperature. The product was collected by filtration, washed with water and air-dried(yield: 45% based on Sm). Yellow block shaped crystals suitable for X-ray analysis were obtained.

### Refinement

H atoms bonded to C atoms were positioned geometrically and refined as riding, with C—H = 0.93 or 0.96 Å and *U*_iso_(H) = 1.2 or 1.5 *U*_eq_(C). H atoms of water molecules were found from difference Fourier maps and refined isotropically with a restraint of O—H = 0.82 Å. The perchlorate ion was disordered over two sites with site occupancy factors 0.560 (11) and 0.440 (11), whereas the methyl group of the acetate ligand was disordered over two sites with site occupancy factors 0.53 (5) and 0.47 (5).

### Crystal data

**Table:**

[AgSm(C_6_H_4_NO_2_)_2_(C_2_H_3_O_2_)(H_2_O)]ClO_4_	*F*(000) = 1300
*M**_r_* = 678.94	*D*_x_ = 2.315 Mg m^−^^3^
Monoclinic, *P*2_1_/*c*	Mo *K*α radiation, λ = 0.71073 Å
Hall symbol: -P 2ybc	Cell parameters from 3844 reflections
*a* = 16.1703 (15) Å	θ = 2.5–27.3°
*b* = 15.1042 (14) Å	µ = 4.19 mm^−^^1^
*c* = 7.9858 (7) Å	*T* = 296 K
β = 92.845 (1)°	Block, yellow
*V* = 1948.0 (3) Å^3^	0.22 × 0.20 × 0.19 mm
*Z* = 4	

### Data collection

**Table:**

Bruker APEXII area-detector diffractometer	3512 independent reflections
Radiation source: fine-focus sealed tube	2957 reflections with *I* > 2σ(*I*)
graphite	*R*_int_ = 0.030
φ and ω scan	θ_max_ = 25.2°, θ_min_ = 1.9°
Absorption correction: multi-scan (*SADABS*; Sheldrick, 1996)	*h* = −17→19
*T*_min_ = 0.414, *T*_max_ = 0.451	*k* = −17→18
9927 measured reflections	*l* = −7→9

### Refinement

**Table:**

Refinement on *F*^2^	Primary atom site location: structure-invariant direct methods
Least-squares matrix: full	Secondary atom site location: difference Fourier map
*R*[*F*^2^ > 2σ(*F*^2^)] = 0.026	Hydrogen site location: inferred from neighbouring sites
*wR*(*F*^2^) = 0.064	H atoms treated by a mixture of independent and constrained refinement
*S* = 1.07	*w* = 1/[σ^2^(*F*_o_^2^) + (0.0316*P*)^2^ + 0.0621*P*] where *P* = (*F*_o_^2^ + 2*F*_c_^2^)/3
3512 reflections	(Δ/σ)_max_ = 0.001
320 parameters	Δρ_max_ = 0.85 e Å^−^^3^
158 restraints	Δρ_min_ = −0.59 e Å^−^^3^

### Special details

**Table:**

Geometry. All e.s.d.'s (except the e.s.d. in the dihedral angle between two l.s. planes) are estimated using the full covariance matrix. The cell e.s.d.'s are taken into account individually in the estimation of e.s.d.'s in distances, angles and torsion angles; correlations between e.s.d.'s in cell parameters are only used when they are defined by crystal symmetry. An approximate (isotropic) treatment of cell e.s.d.'s is used for estimating e.s.d.'s involving l.s. planes.
Refinement. Refinement of *F*^2^ against ALL reflections. The weighted *R*-factor *wR* and goodness of fit *S* are based on *F*^2^, conventional *R*-factors *R* are based on *F*, with *F* set to zero for negative *F*^2^. The threshold expression of *F*^2^ > σ(*F*^2^) is used only for calculating *R*-factors(gt) *etc*. and is not relevant to the choice of reflections for refinement. *R*-factors based on *F*^2^ are statistically about twice as large as those based on *F*, and *R*- factors based on ALL data will be even larger.

### Fractional atomic coordinates and isotropic or equivalent isotropic displacement parameters (Å^2^)

**Table:**

	*x*	*y*	*z*	*U*_iso_*/*U*_eq_	Occ. (<1)
Sm1	0.952467 (14)	0.883640 (13)	1.04772 (3)	0.02097 (9)	
Ag1	0.52807 (3)	1.23823 (4)	1.39951 (7)	0.06436 (17)	
C1	0.8319 (3)	0.7509 (3)	0.7966 (5)	0.0243 (10)	
C2	0.7476 (3)	0.7501 (3)	0.8658 (6)	0.0255 (10)	
C3	0.7080 (3)	0.6710 (3)	0.8907 (6)	0.0357 (12)	
H3	0.7332	0.6178	0.8639	0.043*	
C4	0.6304 (3)	0.6710 (3)	0.9558 (7)	0.0465 (14)	
H4	0.6042	0.6171	0.9726	0.056*	
C5	0.6303 (3)	0.8222 (3)	0.9692 (6)	0.0388 (12)	
H5	0.6037	0.8747	0.9948	0.047*	
C6	0.7077 (3)	0.8270 (3)	0.9062 (6)	0.0331 (11)	
H6	0.7328	0.8816	0.8911	0.040*	
C7	0.8665 (3)	1.0831 (3)	1.1375 (7)	0.0367 (12)	
C8	0.7883 (3)	1.1243 (3)	1.1940 (6)	0.0310 (11)	
C9	0.7177 (3)	1.0739 (3)	1.2021 (7)	0.0414 (13)	
H9	0.7186	1.0143	1.1728	0.050*	
C10	0.6466 (4)	1.1114 (4)	1.2534 (8)	0.0529 (16)	
H10	0.5991	1.0767	1.2566	0.064*	
C11	0.7115 (3)	1.2455 (3)	1.2903 (8)	0.0475 (15)	
H11	0.7096	1.3048	1.3216	0.057*	
C12	0.7843 (3)	1.2127 (3)	1.2376 (7)	0.0431 (14)	
H12	0.8304	1.2490	1.2310	0.052*	
O1	0.8543 (2)	0.68433 (19)	0.7172 (4)	0.0336 (8)	
O2	0.87650 (19)	0.81870 (18)	0.8220 (4)	0.0288 (7)	
O3	0.8699 (2)	1.0005 (2)	1.1391 (5)	0.0493 (11)	
O4	0.9236 (2)	1.1345 (2)	1.0941 (5)	0.0472 (11)	
O5	0.9496 (2)	0.99873 (19)	0.8377 (4)	0.0375 (8)	
O6	0.9636 (2)	1.1151 (2)	0.6844 (4)	0.0400 (9)	
N1	0.5915 (3)	0.7460 (3)	0.9955 (6)	0.0420 (11)	
N2	0.6427 (3)	1.1970 (3)	1.2995 (6)	0.0467 (12)	
O1W	0.9949 (3)	0.72899 (19)	1.0725 (4)	0.0411 (9)	
H2W	1.012 (3)	0.698 (2)	0.999 (5)	0.062*	
H1W	0.974 (3)	0.696 (2)	1.138 (5)	0.062*	
C13	0.9284 (3)	1.0421 (3)	0.7094 (6)	0.0305 (11)	
C14	0.8508 (14)	1.0157 (19)	0.608 (3)	0.048 (4)	0.47 (5)
H14A	0.8496	0.9525	0.5953	0.071*	0.47 (5)
H14B	0.8506	1.0432	0.5000	0.071*	0.47 (5)
H14C	0.8031	1.0345	0.6656	0.071*	0.47 (5)
C14'	0.8691 (14)	1.0029 (16)	0.577 (3)	0.048 (4)	0.53 (5)
H14D	0.8380	1.0496	0.5221	0.071*	0.53 (5)
H14E	0.8318	0.9631	0.6293	0.071*	0.53 (5)
H14F	0.8997	0.9712	0.4966	0.071*	0.53 (5)
Cl1	0.58196 (10)	0.45830 (10)	0.2449 (2)	0.0586 (4)	0.440 (11)
O7	0.5413 (12)	0.5414 (9)	0.242 (2)	0.091 (8)	0.440 (11)
O8	0.6274 (10)	0.4512 (9)	0.3969 (14)	0.107 (6)	0.440 (11)
O9	0.5293 (9)	0.3894 (8)	0.216 (2)	0.123 (6)	0.440 (11)
O10	0.6425 (9)	0.4616 (9)	0.1148 (18)	0.124 (6)	0.440 (11)
Cl1'	0.58196 (10)	0.45830 (10)	0.2449 (2)	0.0586 (4)	0.560 (11)
O7'	0.5434 (8)	0.5371 (7)	0.201 (2)	0.080 (5)	0.560 (11)
O8'	0.6654 (5)	0.4696 (6)	0.2946 (18)	0.101 (4)	0.560 (11)
O9'	0.5408 (9)	0.4196 (8)	0.3857 (15)	0.139 (6)	0.560 (11)
O10'	0.5760 (8)	0.3951 (8)	0.1174 (13)	0.116 (5)	0.560 (11)

### Atomic displacement parameters (Å^2^)

**Table:**

	*U*^11^	*U*^22^	*U*^33^	*U*^12^	*U*^13^	*U*^23^
Sm1	0.02057 (14)	0.01549 (13)	0.02745 (14)	−0.00121 (9)	0.00717 (9)	−0.00001 (9)
Ag1	0.0295 (3)	0.0884 (4)	0.0776 (4)	0.0140 (2)	0.0260 (2)	−0.0008 (3)
C1	0.023 (3)	0.024 (2)	0.026 (2)	−0.0023 (19)	0.0013 (19)	0.0000 (19)
C2	0.023 (3)	0.026 (2)	0.028 (3)	−0.0038 (19)	0.0054 (19)	−0.0029 (19)
C3	0.031 (3)	0.028 (3)	0.049 (3)	−0.004 (2)	0.013 (2)	−0.005 (2)
C4	0.034 (3)	0.041 (3)	0.066 (4)	−0.010 (3)	0.011 (3)	0.002 (3)
C5	0.026 (3)	0.040 (3)	0.051 (3)	0.008 (2)	0.012 (2)	0.002 (2)
C6	0.029 (3)	0.024 (2)	0.047 (3)	0.002 (2)	0.014 (2)	0.001 (2)
C7	0.031 (3)	0.025 (2)	0.056 (3)	0.001 (2)	0.019 (2)	0.002 (2)
C8	0.027 (3)	0.030 (2)	0.037 (3)	0.005 (2)	0.013 (2)	0.003 (2)
C9	0.034 (3)	0.028 (3)	0.063 (4)	−0.005 (2)	0.017 (3)	−0.004 (3)
C10	0.031 (3)	0.047 (3)	0.083 (5)	−0.009 (3)	0.019 (3)	0.005 (3)
C11	0.035 (3)	0.034 (3)	0.075 (4)	0.006 (2)	0.021 (3)	−0.009 (3)
C12	0.029 (3)	0.028 (3)	0.073 (4)	0.000 (2)	0.017 (3)	−0.003 (3)
O1	0.0282 (19)	0.0250 (16)	0.049 (2)	−0.0004 (14)	0.0150 (15)	−0.0096 (15)
O2	0.0237 (18)	0.0259 (16)	0.0371 (19)	−0.0067 (14)	0.0060 (14)	−0.0072 (14)
O3	0.044 (2)	0.0207 (17)	0.086 (3)	0.0039 (16)	0.038 (2)	0.0031 (17)
O4	0.039 (2)	0.0222 (17)	0.083 (3)	0.0019 (15)	0.036 (2)	0.0050 (17)
O5	0.050 (2)	0.0264 (16)	0.0346 (19)	−0.0114 (16)	−0.0101 (16)	0.0089 (15)
O6	0.053 (3)	0.0338 (18)	0.033 (2)	−0.0138 (17)	−0.0029 (16)	0.0100 (15)
N1	0.026 (3)	0.048 (3)	0.054 (3)	−0.004 (2)	0.014 (2)	0.002 (2)
N2	0.028 (3)	0.046 (3)	0.067 (3)	0.011 (2)	0.017 (2)	0.003 (2)
O1W	0.065 (3)	0.0229 (17)	0.037 (2)	0.0084 (18)	0.0223 (19)	0.0043 (15)
C13	0.035 (3)	0.030 (2)	0.026 (3)	−0.005 (2)	0.001 (2)	0.001 (2)
C14	0.047 (6)	0.048 (6)	0.048 (6)	−0.008 (5)	−0.007 (5)	0.003 (4)
C14'	0.047 (6)	0.048 (6)	0.048 (6)	−0.008 (5)	−0.007 (5)	0.003 (4)
Cl1	0.0610 (11)	0.0449 (8)	0.0692 (11)	0.0000 (8)	−0.0030 (8)	−0.0018 (8)
O7	0.097 (11)	0.085 (10)	0.090 (10)	0.023 (8)	0.000 (7)	−0.016 (7)
O8	0.130 (10)	0.104 (8)	0.083 (8)	0.032 (8)	−0.039 (7)	−0.001 (7)
O9	0.129 (10)	0.092 (8)	0.143 (11)	−0.067 (7)	−0.032 (8)	0.025 (7)
O10	0.130 (10)	0.120 (9)	0.125 (10)	0.036 (8)	0.045 (8)	0.001 (7)
Cl1'	0.0610 (11)	0.0449 (8)	0.0692 (11)	0.0000 (8)	−0.0030 (8)	−0.0018 (8)
O7'	0.071 (7)	0.071 (7)	0.100 (8)	0.030 (5)	0.024 (6)	0.043 (6)
O8'	0.065 (6)	0.079 (6)	0.158 (9)	−0.003 (5)	−0.016 (6)	−0.012 (6)
O9'	0.164 (10)	0.131 (8)	0.128 (8)	−0.020 (7)	0.068 (7)	0.039 (7)
O10'	0.127 (9)	0.122 (8)	0.097 (7)	0.022 (7)	−0.017 (6)	−0.058 (6)

### Geometric parameters (Å, °)

**Table:**

Sm1—O2	2.345 (3)	C9—C10	1.364 (7)
Sm1—O3	2.352 (3)	C9—H9	0.9300
Sm1—O4^i^	2.367 (3)	C10—N2	1.346 (6)
Sm1—O1^ii^	2.371 (3)	C10—H10	0.9300
Sm1—O5	2.414 (3)	C11—N2	1.337 (7)
Sm1—O1W	2.440 (3)	C11—C12	1.364 (7)
Sm1—O6^i^	2.476 (3)	C11—H11	0.9300
Sm1—O5^i^	2.521 (3)	C12—H12	0.9300
Sm1—C13^i^	2.892 (5)	O1—Sm1^iv^	2.371 (3)
Sm1—Sm1^i^	3.9263 (5)	O4—Sm1^i^	2.367 (3)
Ag1—N2	2.148 (4)	O5—C13	1.249 (5)
Ag1—N1^iii^	2.149 (4)	O5—Sm1^i^	2.521 (3)
C1—O1	1.252 (5)	O6—C13	1.262 (5)
C1—O2	1.264 (5)	O6—Sm1^i^	2.476 (3)
C1—C2	1.495 (6)	N1—Ag1^v^	2.149 (4)
C2—C3	1.375 (6)	O1W—H2W	0.81 (4)
C2—C6	1.375 (6)	O1W—H1W	0.81 (4)
C3—C4	1.382 (7)	C13—C14	1.511 (10)
C3—H3	0.9300	C13—C14'	1.511 (9)
C4—N1	1.341 (6)	C13—Sm1^i^	2.892 (5)
C4—H4	0.9300	C14—H14A	0.9600
C5—N1	1.333 (6)	C14—H14B	0.9600
C5—C6	1.373 (6)	C14—H14C	0.9600
C5—H5	0.9300	C14'—H14D	0.9600
C6—H6	0.9300	C14'—H14E	0.9600
C7—O3	1.248 (5)	C14'—H14F	0.9600
C7—O4	1.267 (6)	Cl1—O9	1.357 (9)
C7—C8	1.500 (6)	Cl1—O8	1.391 (9)
C8—C9	1.376 (7)	Cl1—O7	1.417 (10)
C8—C12	1.381 (6)	Cl1—O10	1.464 (9)
			
O2—Sm1—O3	105.57 (13)	C5—C6—H6	120.4
O2—Sm1—O4^i^	90.42 (12)	C2—C6—H6	120.4
O3—Sm1—O4^i^	137.99 (11)	O3—C7—O4	125.7 (4)
O2—Sm1—O1^ii^	85.29 (11)	O3—C7—C8	116.7 (4)
O3—Sm1—O1^ii^	75.00 (10)	O4—C7—C8	117.6 (4)
O4^i^—Sm1—O1^ii^	146.13 (10)	C9—C8—C12	118.3 (4)
O2—Sm1—O5	77.05 (10)	C9—C8—C7	119.9 (4)
O3—Sm1—O5	71.54 (12)	C12—C8—C7	121.8 (4)
O4^i^—Sm1—O5	74.83 (12)	C10—C9—C8	119.9 (5)
O1^ii^—Sm1—O5	135.91 (12)	C10—C9—H9	120.1
O2—Sm1—O1W	78.25 (13)	C8—C9—H9	120.1
O3—Sm1—O1W	148.79 (11)	N2—C10—C9	122.1 (5)
O4^i^—Sm1—O1W	71.75 (11)	N2—C10—H10	118.9
O1^ii^—Sm1—O1W	74.48 (11)	C9—C10—H10	118.9
O5—Sm1—O1W	137.85 (11)	N2—C11—C12	123.4 (5)
O2—Sm1—O6^i^	155.64 (10)	N2—C11—H11	118.3
O3—Sm1—O6^i^	91.24 (14)	C12—C11—H11	118.3
O4^i^—Sm1—O6^i^	88.48 (14)	C11—C12—C8	118.8 (5)
O1^ii^—Sm1—O6^i^	82.17 (11)	C11—C12—H12	120.6
O5—Sm1—O6^i^	125.83 (10)	C8—C12—H12	120.6
O1W—Sm1—O6^i^	78.31 (12)	C1—O1—Sm1^iv^	148.9 (3)
O2—Sm1—O5^i^	150.36 (10)	C1—O2—Sm1	137.3 (3)
O3—Sm1—O5^i^	73.42 (13)	C7—O3—Sm1	140.5 (3)
O4^i^—Sm1—O5^i^	73.96 (12)	C7—O4—Sm1^i^	134.8 (3)
O1^ii^—Sm1—O5^i^	121.62 (11)	C13—O5—Sm1	160.4 (3)
O5—Sm1—O5^i^	74.62 (12)	C13—O5—Sm1^i^	94.0 (3)
O1W—Sm1—O5^i^	118.44 (13)	Sm1—O5—Sm1^i^	105.38 (12)
O6^i^—Sm1—O5^i^	51.22 (10)	C13—O6—Sm1^i^	95.9 (3)
O2—Sm1—C13^i^	169.71 (12)	C5—N1—C4	117.6 (4)
O3—Sm1—C13^i^	82.55 (14)	C5—N1—Ag1^v^	123.2 (3)
O4^i^—Sm1—C13^i^	79.29 (14)	C4—N1—Ag1^v^	119.2 (3)
O1^ii^—Sm1—C13^i^	103.16 (12)	C11—N2—C10	117.5 (4)
O5—Sm1—C13^i^	100.12 (12)	C11—N2—Ag1	126.6 (4)
O1W—Sm1—C13^i^	98.16 (14)	C10—N2—Ag1	115.6 (4)
O6^i^—Sm1—C13^i^	25.73 (11)	Sm1—O1W—H2W	127 (3)
O5^i^—Sm1—C13^i^	25.53 (11)	Sm1—O1W—H1W	121 (3)
O2—Sm1—Sm1^i^	114.86 (7)	H2W—O1W—H1W	106 (4)
O3—Sm1—Sm1^i^	67.80 (8)	O5—C13—O6	118.7 (4)
O4^i^—Sm1—Sm1^i^	70.22 (7)	O5—C13—C14	119.1 (11)
O1^ii^—Sm1—Sm1^i^	141.10 (7)	O6—C13—C14	121.0 (11)
O5—Sm1—Sm1^i^	38.26 (8)	O5—C13—C14'	120.4 (10)
O1W—Sm1—Sm1^i^	139.62 (10)	O6—C13—C14'	120.5 (10)
O6^i^—Sm1—Sm1^i^	87.58 (7)	C14—C13—C14'	16.7 (16)
O5^i^—Sm1—Sm1^i^	36.36 (7)	O5—C13—Sm1^i^	60.4 (2)
C13^i^—Sm1—Sm1^i^	61.87 (9)	O6—C13—Sm1^i^	58.4 (2)
N2—Ag1—N1^iii^	166.24 (17)	C14—C13—Sm1^i^	165.6 (14)
O1—C1—O2	123.7 (4)	C14'—C13—Sm1^i^	177.6 (12)
O1—C1—C2	118.2 (4)	C13—C14—H14A	109.5
O2—C1—C2	118.1 (4)	C13—C14—H14B	109.5
C3—C2—C6	118.2 (4)	C13—C14—H14C	109.5
C3—C2—C1	120.0 (4)	C13—C14'—H14D	109.5
C6—C2—C1	121.8 (4)	C13—C14'—H14E	109.5
C2—C3—C4	119.6 (4)	H14D—C14'—H14E	109.5
C2—C3—H3	120.2	C13—C14'—H14F	109.5
C4—C3—H3	120.2	H14D—C14'—H14F	109.5
N1—C4—C3	122.2 (5)	H14E—C14'—H14F	109.5
N1—C4—H4	118.9	O9—Cl1—O8	112.8 (7)
C3—C4—H4	118.9	O9—Cl1—O7	113.0 (9)
N1—C5—C6	123.2 (4)	O8—Cl1—O7	108.0 (8)
N1—C5—H5	118.4	O9—Cl1—O10	110.1 (8)
C6—C5—H5	118.4	O8—Cl1—O10	106.2 (8)
C5—C6—C2	119.3 (4)	O7—Cl1—O10	106.5 (8)

Symmetry codes: (i) −*x*+2, −*y*+2, −*z*+2; (ii) *x*, −*y*+3/2, *z*+1/2; (iii) −*x*+1, *y*+1/2, −*z*+5/2; (iv) *x*, −*y*+3/2, *z*−1/2; (v) −*x*+1, *y*−1/2, −*z*+5/2.

### Hydrogen-bond geometry (Å, °)

**Table:**

*D*—H···*A*	*D*—H	H···*A*	*D*···*A*	*D*—H···*A*
O1W—H2W···O6^vi^	0.81 (4)	1.98 (4)	2.785 (4)	171 (6)
O1W—H1W···O2^ii^	0.81 (4)	2.22 (3)	2.921 (5)	146 (5)

Symmetry codes: (vi) −*x*+2, *y*−1/2, −*z*+3/2; (ii) *x*, −*y*+3/2, *z*+1/2.
